# Machine learning analysis of ARVC informed by sodium channel protein-based interactome networks

**DOI:** 10.3389/fphar.2025.1611342

**Published:** 2025-07-23

**Authors:** Yanan Zhu, Hui Zhang, Xuan Zhao, Xin Wang, Lina Xing, Sijie Yao, Xiao Jin, Tingting Li, Ting Dai, Xinyue Ding, Zhen Qi, Zongjun Liu

**Affiliations:** ^1^ Department of Cardiology, Putuo Hospital, Shanghai University of Traditional Chinese Medicine, Shanghai, China; ^2^ Shanghai Key Laboratory of Regulatory Biology, School of Life Sciences, East China Normal University, Shanghai, China; ^3^ Department of GCP Office, Shanghai Ninth People’s Hospital, Shanghai Jiao Tong University School of Medicine, Shanghai, China; ^4^ Neocellmed Co., Ltd., Shanghai, China

**Keywords:** ARVC, sodium channel protein, machine learning, protein-protein interaction, binding affinity, cardiac organoids

## Abstract

**Background:**

Arrhythmogenic right ventricular cardiomyopathy (ARVC) is an inherited cardiac disorder characterized by sodium channel dysfunction. However, the clinical management of ARVC remains challenging. Identifying novel compounds for the treatment of ARVC is crucial for advancing drug development.

**Purpose:**

In this study, we aim to identify novel compounds for treating ARVC.

**Methods:**

Machine learning (ML) models were constructed using proteins analyzed from the scRNA-seq data of ARVC rats and their corresponding protein-protein interaction (PPI) network to predict binding affinity (BA). To validate these predictions, a series of experiments in cardiac organoids were conducted, including Western blotting, ELISA, MEA, and Masson staining to assess the effects of these compounds.

**Results:**

We first discovered and identified SCN5A as the most significantly affected sodium channel protein in ARVC. ML models predicted that Kaempferol binds to SCN5A with high affinity. *In vitro* experiments further confirmed that Kaempferol exerted therapeutic effects in ARVC.

**Conclusion:**

This study presents a novel approach for identifying potential compounds to treat ARVC. By integrating ML modeling with organoid validation, our platform provides valuable support in addressing the public health challenges posed by ARVC, with broad application prospects. Kaempferol shows promise as a lead compound for ARVC treatment.

## Graphical Abstract

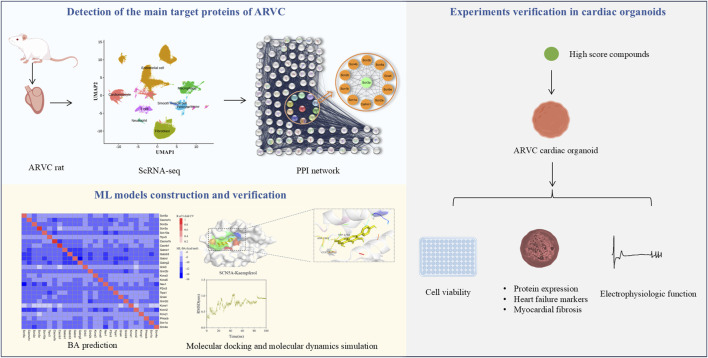

## 1 Introduction

Arrhythmogenic right ventricular cardiomyopathy (ARVC) is an inherited cardiac disorder with a prevalence ranging from 1:2000 to 1:5000, predominantly affecting males (Pilichou et al., 2016). The structural defects of ARVC are characterized by fibrofatty infiltration and replacement of the myocardium, leading to ventricular dysfunction, life-threatening ventricular arrhythmias, and heart failure (Basso et al., 2012). As a familial susceptibility disease, mutations in desmosomal genes are the primary pathogenic factors. Plakophilin 2 (PKP2), has recently been identified as the most commonly affected gene in ARVC, with over 40% of the ARVC population respectively (Austin et al., 2019). Previous studies indicate that PKP2 mutations are strongly associated with sodium channel dysfunction (Cerrone and Delmar, 2014; Shaw, 2013). Therefore, focusing on ARVC associated with PKP2 mutations and sodium channel alterations is of considerable importance.

The clinical management of ARVC remains challenging. Current strategies primarily focus on symptomatic relief or lifestyle modifications, including exercise restriction, pharmacological interventions, and invasive measures such as catheter ablation, implantable cardioverter-defibrillator, and heart transplantation (Sayed et al., 2020; Krahn et al., 2022). While antiarrhythmic drugs are commonly used, their therapeutic efficacy remains suboptimal. In recent years, gene therapy, particularly adeno-associated virus vector (AAV) - mediated gene therapy, has garnered significant attention. However, biosafety concerns must be carefully addressed (Wilton-Clark and Yokota, 2023). To date, no approved therapies exist to treat myocardial fibrosis in ARVC (Krahn et al., 2022; Al-Aidarous et al., 2024). However, due to the complexity of the disease and the limited size of patient cohorts, exploring compounds to treat ARVC presents significant challenges.

The protein-protein interaction (PPI) network consists of proteins and their interactions, providing a foundation for systematically investigating potential treatment efficacy and contributing to specific biological functions. PPI networks related to major sodium channel proteins can facilitate systematic investigations into drug treatments (Szklarczyk et al., 2019). However, traditional experimental methods for testing the targets within these PPI networks are time-consuming and costly (Jia et al., 2009). Recently, network-based approaches have been increasingly utilized in drug discovery, and machine learning (ML) technologies have gained significant traction in drug discovery and development (Gao et al., 2020a). These tools enable computational prioritization of high-value compounds, thereby substantially reducing both time and costs while addressing ethical concerns (Gao et al., 2020b; Qiu and Wei, 2023). Consequently, ML approaches were employed in this study to perform large-scale predictions.

The present study constructed an ML platform to identify the therapeutic compounds of ARVC. The PPI networks of the 8 major sodium channel proteins identified in ScRNA-seq were obtained from the STRING database. Potential compounds with experimentally determined binding affinities were sourced from the ChEMBL database, and ML models were constructed. Compounds were represented by molecular fingerprints using SMILES Transformer and paired with the gradient-boosting decision tree (GBDT) algorithm to develop binding affinity (BA) predictors. These compounds were subsequently tested in ARVC cardiac organoids. Our platform holds promise for advancing drug development for ARVC treatment.

## 2 Materials and methods

### 2.1 Generation of PKP2^+/−^ animals

The PKP2 mutation (PKP2^+/−^) rat as well as their wild type (WT) littermates (30-week-old) were obtained from Prof. Wang Xin (East China Normal University, Shanghai, China). All rats were fed in the Laboratory Animal Center of Putuo Hospital, Shanghai University of Traditional Chinese Medicine. The environment was set to maintain a relative humidity of 45%–55% under a controlled room temperature of 22°C–24°C with a 12–12 h light-dark cycle.

### 2.2 ECG and Magnetic Resonance Imaging (MRI)

ECG was conducted as previously described (Bradford et al., 2023). Briefly, rats were anesthetized with pentobarbital (20 mg/kg) during the experiment, and the chest hair was removed. Rats (30 weeks old) were placed on a heated pad and fixed in a dorsal position. Needle electrodes were inserted subcutaneously into the limbs. Electrical activity was recorded by the IX-BIO4 system (iWorx Systems, Inc., USA) for 20 min. The MRI was conducted on a BioSpec 94/30 USR (Bruker BioSpin MRI GMBH, Germany) as previously described (Liang et al., 2021).

The animal experiments were performed according to the Guidelines for Care and Use of Laboratory Animals. All experiments were approved by the Animal Ethic Committee of Putuo Hospital (approved number: DWEC-A-22024-02-2-72).

### 2.3 Protein analysis

Western blot analysis was performed as previously described. Oridonin (ODN) (ChemFaces, 28957-04-2) or Kaempferol (KA) (Aladdin, 520-18-3) at indicated concentrations (5 μM, 10 μM, 20 μM or 1 μM, 3 μM) was added to the PKP2^+/−^ cardiac organoids and incubated for 48 h. The cardiac organoids were then collected and washed in cold sterile PBS and the total protein extract was isolated via the protein extraction kit (KeyGEN BioTECH, China).

Immunodetection of PKP2 (Abcam, ab223757), Nav1.5 (SCN5A) (CST, 14421), and GAPDH (Abcam, ab181602) was done as previously described (Lyon et al., 2014). Briefly, the cardiac organoids’ proteins were loaded onto the 12% SDS-polyacrylamide gel electrophoresis (SDS-PAGE) gels and then transferred to PVDF membranes. After being blocked in 5% non-fat milk in TBS-T at room temperature for 1 h, the membranes were incubated overnight at 4°C with primary antibodies. Then, the HRP-labeled goat anti-rabbit secondary antibody (H + L) (Beyotime, A0208) was used. The Clarity Western ECL Substrate kit was used for visualization. Tanon-4600 and ImageJ software (NIH, Bethesda, MD) were used to analyze the immunoblots.

### 2.4 Histological analysis

The rat hearts or cardiac organoids were fixed with 4% PFA (Servicebio, G1101) overnight and were embedded in the O.C.T. Compound (Sakura, 4583). Then, the frozen sections were cut to 5 µm thickness and restored to room temperature. Next, the slices were stained with the Masson dye solution set (Servicebio, G1006) according to the manufacturer’s instructions. Images were acquired with the NIKON ECLIPSE E100.

### 2.5 Single-cell RNA-sequence (scRNA-seq)

#### 2.5.1 Preparation of the single cardiac cell suspension

Fresh 3 cardiac tissue was collected from each group with a surgical scissor to isolate single cells. The tissues were cut into small pieces of 0.5 mm^3^ in prechilled PBS with surgical scissors and digested with collagenase type II (Gibco, 17101015) in a constant temperature incubator at 37 °C and mixed inversely every 5 min. The digested cell suspension was filtered with a 40 μm cell screen (Falcon, 352340) 2 times and centrifuged at 4°C and 300 g for 5 min. The supernatant was discarded, and the precipitate was resuspended in 100 μL of 10% FBS/DMEM, and the cell concentration and viability were calculated by Luna cell counter. Dead cell removal was performed according to the MACS Dead Cell Removal Kit (MACS, 130-090-101) operating instructions. In brief, the cell suspension was added with 100 μL of magnetic beads and incubated at room temperature for 15 min. Then the suspension was passed through the column. The eluted cell suspension was centrifuged at 4°C and 300 g for 5 min and resuspended with 100 μL medium, and detected by Luna cell counter (Logos Biosystems, Korea).

#### 2.5.2 Single-cell transcriptome capture, RNA-Seq library construction, and sequence

The single-cell suspension was adjusted to 700–1200 cells/μL according to the 10 × Genomics Chromium Next GEM Single Cell 3ʹ Reagent Kits v3.1 (No.1000268) Operation manual for computer and library construction. The constructed library was sequenced using the Illumina Nova 6000 PE150 platform.

#### 2.5.3 Single-cell RNA-Seq data analysis

Seurat software was used for clustering and visualization processing. Firstly, the gene expression matrix of each sample was read and converted into a Seurat object. In the subsequent analysis, the cells were excluded with mitochondrial UMI (Unique Molecular Identifier) which accounted for more than 35% and fewer than 50 genes. After logarithmic normalization based on the total UMI count of cells, the data was scaled according to the UMI count, and principal component analysis (PCA) was performed based on the top 2000 highly variable features. Subsequently, the data was clustered and visualized by Unified Manifold Approximation and Projection (UMAP) under the resolution of 0.5. Feature plots were used to visualize the expression of specific genes in each cluster.

#### 2.5.4 Cell annotation

The FindAllMarkers function combined with the Wilcoxon test was used to calculate the specific markers for each cell cluster under the following criteria: log2 fold change >0.25, min. pct >0.25. A large amount of transcriptome data in Cell Taxonomy (https://ngdc.cncb.ac.cn/celltaxonomy/) and CellMarker (http://117.50.127.228/CellMarker/) were used to annotate cell types for each cell cluster.

### 2.6 Machine-learning analysis

#### 2.6.1 Datasets

The compound database was collected from the CHEMBL database to investigate the proteins with major changes in sodium channels. Since machine-learning models largely rely on a sufficient set of data points, we require the size of the collected dataset to be at least 100. A total of 27 datasets were then obtained. IC_50_ was used as the data point to calculate binding affinity (BA), with the formula $BA=1.3633\times \log10\left(\frac{IC50}{2}\right)$ (kcal/mol), and was then used to construct machine learning models as previously described (Feng et al., 2023; Kalliokoski et al., 2013).

#### 2.6.2 Molecular embeddings

The molecular representation of the 27 collected datasets is 2D SMILES strings. The molecular fingerprints were used to build machine-learning models in this study. SMILES Transformer (ST) is an unsupervised pre-training method that can learn the syntax and semantic information in SMILES strings (Mswahili and Jeong, 2024; Honda et al., 2019). The molecular fingerprints were generated by pre-trained models based on ST and sequence-to-sequence autoencoder (Winter et al., 2019). The model built an encoder-decoder network with 4 Transformer blocks for each with PyTorch. Each Transformer block has 4-head attentions with 256 embedding dimensions and 2 linear layers. Pre-training of ST was performed by 861000 unlabeled smiles randomly sampled in ChEMBL24. A 1024-dimensional fingerprint for each molecule was obtained through the maximum pooling output of the transformer model. By constructing the sequence-to-sequence language model, the efficiency and accuracy of molecular characterization were highly improved.

#### 2.6.3 Machine-learning models

The gradient boosting decision tree (GBDT) algorithm version 0.24.1. (Scikit-learn library), was deployed to build our machine-learning models. The GBDT algorithm can effectively combine the decision tree with the integration idea with the advantage of robustness against overfitting, insensitiveness to hyperparameters, and ease of implementation. Through the bootstrap method for resampling, GBDT generated multiple decision trees and combined the output of these decision trees through the integration method to reduce error.

We collected 27 datasets with at least 100 data points in each dataset. It is preferable to utilize GBDT in building machine-learning models for these datasets. As previously described, molecular fingerprints generated by SMILES Transformer were adopted to represent compounds. The 27 machine-learning models were constructed by integrating molecular fingerprints with the GBDT algorithm. To alleviate the effect of randomness, each GBDT model was trained five times with different random seeds. The average of the five predictions (Pearson correlation coefficients and RMSE) was regarded as the final result of each model.

#### 2.6.4 Molecular docking

The PDB database (https://www.rcsb.org/) was used to obtain the protein’s three-dimensional structure (resolution<3A), and the TCMSP database (https://old.tcmsp-e.com/tcmsp.php) was used to obtain the active ingredient’s three-dimensional structure. The water molecules and tiny molecular linkages were then eliminated using the “PyMOL” program. Using the “AutoDockTools” program, protein and medication ingredients were transformed into PDBQT format files, allowing for the identification of active pockets. Lastly, molecular docking was performed using the “vina” software. The 3D binding interactions of co-crystal structures created by receptors interacting to ligands were depicted using PyMOL.

#### 2.6.5 Molecular dynamic simulation

To explore the stability of the protein–ligand interactions in greater depth, molecular dynamics (MD) simulations were performed on two complexes: SCN5A-Kaempferol, SCN5A-Oridonin, using GROMACS 2024.3 software. The AMBER99SB force field and SPC water model were utilized, with the system temperature set at 300 K and the simulation time at 100 ns. The energy minimization phase employed the steepest descent method, followed by energy equilibration to stabilize the system before completing the MD simulation.

### 2.7 Cardiac organoid culture

The WT and PKP2^+/−^ iPSCs were generated in collaboration with Cellapy (Beijing, China). The differentiation of cardiac organoids started when iPSCs reached 80 ∼ 95% of confluency. The iPSCs were digested by Gentle Cell Dissociation Reagent (STEMCELL Technologies, 07174), and were seeded into a U-shaped ultralow-attachment 96-well plate (Corning, 7007) in mTeSR Plus medium (STEMCELL Technologies, 05825) supplemented with 10 μM Rho kinase inhibitor Y-27632 (Beyotime, SC0326) at a density of 9000 cells per well. The plate was centrifuged at 300 *g* for 5 min. After 24 h incubation, the medium was changed into differentiation medium A, which contains CHIR-99021, BMP-4, LY294002, Activin A, and insulin. 48 h later, the differentiation medium B containing BMP4, IWR1, Retinoic acid, FGF2, and insulin was replaced. 4 days later, differentiation medium C contained FGF2, and insulin was replaced daily for 5 days. Lastly, the organoids were cultured in a maintenance medium supplemented with insulin, and the medium was replaced daily.

### 2.8 Immunofluorescence (IF) analysis

Organoids were fixed with 4% PFA at 4°C overnight. After O.C.T. embedding, the slides were cut to 5 µm thickness and immersed in the EDTA antigen retrieval buffer (Servicebio, G1206) for antigen retrieval. Then, 3% BSA was added to block the non-specific binding for 30 min. After being stained with primary antibody: rabbit anti-alpha smooth muscle (1:500, Abcam, ab5694) or rabbit anti-cTnT (1:200, Abcam, ab209813) overnight at 4°C, the secondary antibody was added and incubated at room temperature for 1 h under dark conditions. Nuclei were stained with DAPI (VECTOR, USA) for 10 min at room temperature. After three times washing with PBS, the images were visualized under a fluorescence microscope (Ortho-Fluorescent Microscopy, Nikon).

### 2.9 Cell viability assay

Cell viability was detected with CCK-8 assay. In brief, cardiac organoids were cultured in U-shape 96-well and incubated with different concentrations of KA or ODN for 48 h. After incubation, the supernatant was discarded, and each well was supplemented with 10% CCK-8 reagent, followed by 1 h incubation at 37°C as previously described (Chen X. et al., 2023). Then, cell viability was measured at 450 nm using a spectrophotometer. Each group had at least five replicates.

### 2.10 MEA assay

The MEA assay was conducted to evaluate the therapeutic effect of the KA on electrophysiological properties in cardiac organoids. Briefly, the WT and PKP2^+/−^ cardiac organoids were seeded into the Matrigel-coated CytoView MEA plates, with one organoid per well for electro-activity recording. After being treated with KA for 48 h, the electro-activities were recorded for 5 min. The MEA data including the Beat Means Periods, Spikes Amplitude, and Field Potential Duration were analyzed and visualized using the Maestro Pro multi-well MEA platform and cardiac analysis tool (Axion BioSystems, USA).

### 2.11 Statistical analysis

All graphs were plotted by GraphPad Prism 9.0 software (GraphPad, Avenida, CA, United States). All data were presented as the means ± S.E.M. (standard error of measurement). Ordinary one-way ANOVA followed by the Dunnett *post hoc* test or Student’s t-test was performed to analyze the differences in variables. *P* < 0.05 was considered statistically significant.

## 3 Result

### 3.1 SCN5A serves as a key element in ARVC with PKP2 mutation

The ARVC rat model was generated using CRISPR-Cas9-mediated genome editing, introducing a frameshift mutation with a 5-base pair (bp) knockout in the PKP2 gene. Founder rats were screened by Sanger sequencing to confirm the precise 5-bp deletion (Supplementary Figure S1A). Next, ECG analysis, MASSON’s staining, Western blotting, and MRI were conducted to confirm the absence of functional PKP2 protein (Supplementary Figures S1B–E). A widened QRS complex and lower PKP2 expression were observed in PKP2^+/−^ rats compared to the littermate WT controls, consistent with previous findings in mice (Kyriakopoulou et al., 2023) (Supplementary Figures S1B,C). Additionally, Masson’s trichrome staining and MRI revealed significant myocardial loss, fibrosis, fatty tissue replacement, and right ventricular (RV) dilation in PKP2^+/−^ rats compared to WT controls (Supplementary Figures S1D,E). Together, these results indicate that the ARVC model has been successfully constructed.

**FIGURE 1:**
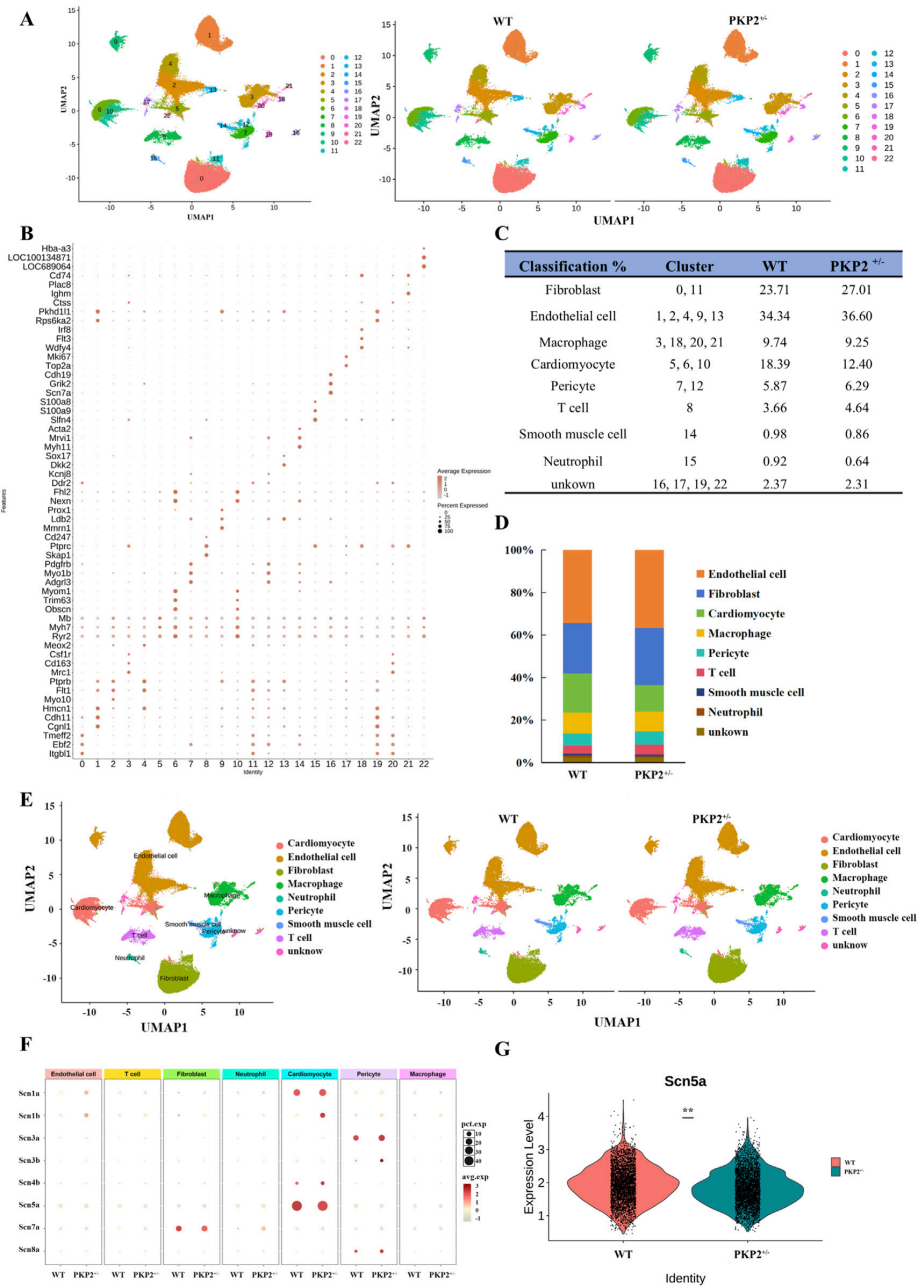
Identification of cell populations of rat cardiac tissue. **(A)** Uniform manifold approximation and projection (UMAP) analysis integrating 54828 single cells from WT and PKP2^+/−^ ventricles. **(B)** Dot plot visualization of top marker genes used to identify clusters. The color and size of the dots represent the relative average expression levels in each population and the percentage of cells expressing the gene, respectively. **(C)** Fraction of cell types. **(D)** Bar plot of the percentage contributions of each cluster in the scRNA-seq samples. **(E)** UMAP of single cells colored by clusters in WT (n = 2 ventricles) and PKP2^+/−^(n = 2 ventricles). Nine clusters represented by colors are marked with the presumed cell types. **(F)** Expression bubble plot of sodium channel-related genes in different cell types. **(G)** Violin plot of Scn5a expression between groups.

The heart tissues were collected from WT and PKP2^+/−^ rats above, digested, and analyzed for ScRNA-seq. The unsupervised clustering of the 32326 cardiac cells using UMPA (Uniform Manifold Approximation and Projection) revealed a high correlation among the 23 obtained clusters (Figure 1A), allowing individual cells to be classified as homogeneous states. According to the significant expression of established lineage markers and representative genes (Figure 1B), the clusters were defined as Fibroblast (cluster 0, 11), Endothelial cell (cluster 1, 2, 4, 9, 13), Macrophage (cluster 3, 18, 20, 21), Cardiomyocyte (cluster 5, 6, 10), Pericyte (cluster 7, 12), T cell (cluster 8), Smooth muscle cell (cluster 14), Neutrophil (cluster 15), unknown (cluster 16,17,19,22) (Figures 1C,D). Each color expressed different cell types (Figure 1E). In addition, in the PKP2^+/−^ group, there was a significant increase in the cluster of fibroblasts, which was in accordance with the previously found in ARVC (Supplementary Figures S1C,D).

The gene analysis of the ScRNA-seq indicated significant differences in sodium channel between WT and PKP2^+/−^ groups (Figure 1F): Fibroblast (Scn1b, Scn4b, Scn3b), Endothelial cell (Scn1b, Scn1a, Scn3a, Scn4b, Scn5a), Macrophage (Scn1b, Scn7a, Scn4b), Cardiomyocyte (Scn1b, Scn4b), Pericyte (Scn1b, Scn3b, Scn8a), T cell (Scn1b), Neutrophil (Scn7a). Next, the differential gene expression between PKP2^+/−^ and WT in the whole tissue was further analyzed. Among the above 8 sodium ion-related genes, only Scn5a exhibited a significant downregulation in the PKP2^+/−^ group (Figure 1F), which was in accordance with the proteomics (Supplementary Figure S2 and Supplementary File S4). In summary, these results suggest that Scn5a may play a crucial role in ARVC.

**FIGURE 2:**
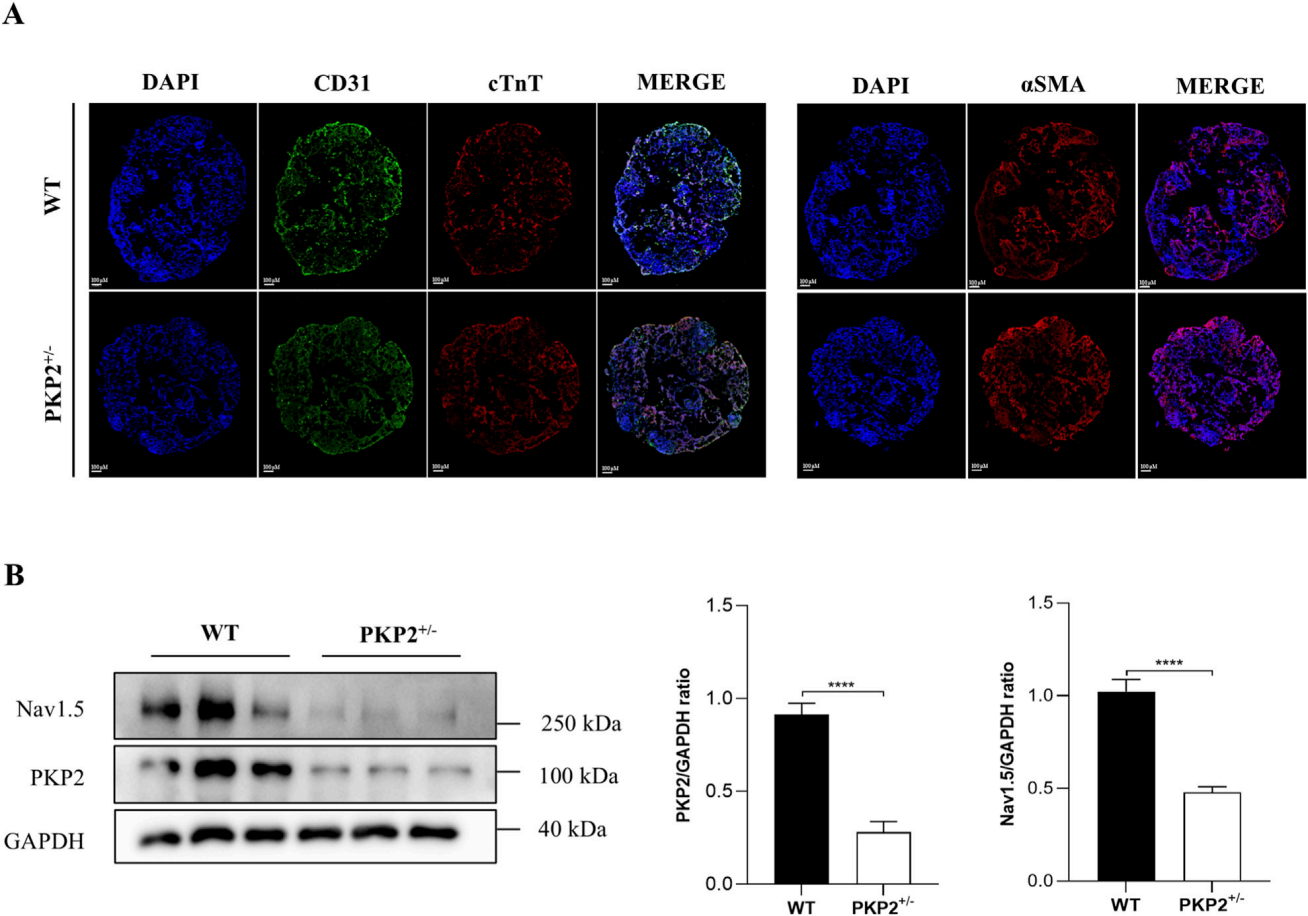
The generation and characterization of WT and PKP2^+/−^ iPSC-derived cardiac organoids. **(A)** Immunofluorescence staining for cardiac cell marker cTnT, endothelial cell marker CD31, and myofibroblast cell marker α-SMA of cardiac organoids. Scale bars, 100 μm. **(B)** Representative immunoblots and quantifications for PKP2 and Nav1.5 in WT and PKP2^+/−^ cardiac organoids. GAPDH was used as a loading control. Two-tailed unpaired t-test. The results are presented as the percentage of means ± S.E.M. (n = 3–6). *P < 0.05, **P < 0.01, ***P < 0.005, ****P < 0.001 vs. Control group.

Next, the human cardiac organoid model was subsequently employed to further validate the findings observed in rats. Firstly, WT and PKP2^+/−^ hiPSCs were induced to undergo directed differentiation into cardiac organoids, and the immunofluorescence staining was conducted to characterize the cardiac organoids. As shown in Figure 2A, the myocardial marker cTnT, the endothelial cell markers CD31, and the fibroblast marker α-SMA were highly expressed in organoids. Then, Western blot analysis was performed to investigate the SCN5A (Nav1.5) expression. The results revealed that PKP2 expression was significantly reduced in PKP2^+/−^ cardiac organoids compared to WT organoids, consistent with previous reports (Kyriakopoulou et al., 2023). Additionally, the expression of SCN5A was aligned with the results from ScRNA-Seq and proteomics in rats (Figure 2B). Taken together, these findings suggest that SCN5A plays a key role in ARVC associated with PKP2 mutations.

### 3.2 Machine-learning analysis of sodium channel proteins-based interactome networks

**FIGURE 3:**
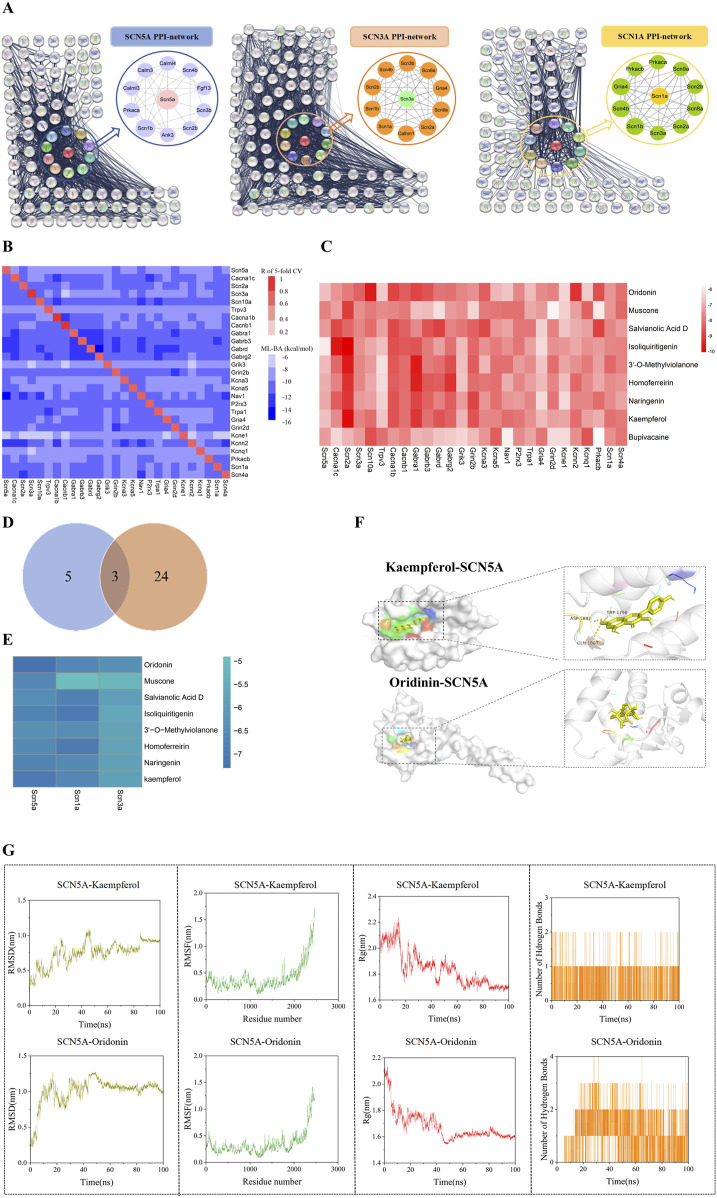
A Protein-protein interaction network of sodium channel-associated genes. **(A)** Protein-protein interaction network of sodium channel-associated genes (Full names of the proteins in the eight core networks are provided in the Supplementary Material). **(B)** The heatmap of cross-target binding affinities (BA) predictions reveals the specificity of each dataset and other protein targets. The notations below the heatmap show the machine-learning models, while those on the right of the heatmap denote all the datasets. The diagonal elements in the heatmap indicate the Pearson correlation efficiency (R) of five-fold cross-validation for all the predictive models. The off-diagonal elements in each row represent the highest BA values of medicine in one predicted by 28 machine-learning models. **(C)** The BA value of the drug predicted by 28 machine-learning models. **(D)** Venn diagram of sodium channel differential gene and machine learning model. **(E)** Binding energy heatmap of molecular docking of proteins (SCN5A, SCN1A, SCN3A) and compounds. Representative docking pattern between SCN5A and optimal binding active compounds. **(F)** Molecular dynamics simulation between SCN5A and optimal binding active compounds. **(G)** Molecular dynamics simulation between SCN5A and optimal binding active compounds. The values of root mean square deviation (RMSD), root mean square fluctuation (RMSF), radius of gyration (Rg), and the number of hydrogen bonds of the two complexes are presented.

Sodium channel proteins and their interactions play a critical role in specific biological functions (Feng et al., 2023; Gao et al., 2021). To ensure a sufficient dataset for machine learning models, we selected the 8 proteins related to sodium ion channels in ScRNA-seq as targets for PPI analysis by inputting these protein names into the STRING database. In each network, there is a core subnetwork with proteins that interact directly with each sodium channel protein, while additional proteins with interactions collectively form the global network as shown in Figure 3A and Supplementary File S1.

To evaluate the binding effect of the potential compounds to sodium channel proteins in the PPI networks, we collected compounds from the ChEMBL database. Next, the machine learning model is established by combining the molecular fingerprint trained by the ST algorithm with cross-target BA predictions. We assembled 27 datasets in total and built 27 ML models, with sufficient data points (>100) for the proteins in the respective PPI networks. The details about the collected datasets can be found in Supplementary File S2. The diagonal elements represented the Pearson correlation coefficient (R) of the 5-fold cross-validation of the Machine-Learning BA predictions for the corresponding protein database. The off-diagonal elements indicated the maximal BA values predicted by other models. The horizontal axis notations corresponded to the 27 datasets, while the vertical axis notations represented the 27 ML models. The intensity of cross-target BA was indicated by the color of off-diagonal elements, with darker shades corresponding to weaker BA. 3 of the 27 models have R values >0.8, with the lowest R-value being 0.62 for the Kcna5 model (Figure 3B). Additionally, the RMSE values of these models fell within a reasonable range of [0.722, 1.686] kcal/mol as shown in Supplementary File S3. Overall, these models demonstrated high prediction accuracy and proved reliable for BA predictions.

### 3.3 Screening potential compounds by using the ML models

There have been no reported compounds with agonist effects on sodium channel proteins in ARVC, hence prompting us to explore potential compounds that may bind to these sodium channel proteins. Since heart failure is a distinct end-stage symptom of ARVC (Lu et al., 2022), we used the above 27 ML models to screen several compounds reported to have therapeutic effects on heart failure (Lin et al., 2022; Iqbal et al., 2023; Du et al., 2018; Chen K. et al., 2023; Xu et al., 2023). The results indicated that the BA values between compounds and sodium channel-related proteins were high, with a mean range of [-7.823, 7.204]. Among them, Kaempferol (KA) had a BA value of −7.823, indicating a strong affinity. Concurrently, we included Bupivacaine, an SCN5A inhibitor (Schwoerer et al., 2015), as a positive control. Its high binding affinity (BA = −7.343 kcal/mol) confirmed successful model validation (Figure 3C). Interestingly, SCN1A, SCN5A, and SCN3A were identified as the overlapping proteins between the 8 differentially expressed genes from scRNA-seq and the 27 sodium ion channel proteins used in the ML model construction (Figure 3D). To further assess the interactions, molecular docking was performed to evaluate the binding energy between the compounds and these proteins. The result showed higher binding energy between Kaempferol (KA), Oridonin (ODN), and SCN5A (Figure 3E). The interaction diagram of molecules and proteins shows that KA is hydrogen-bonded to SCN5A (Figure 3F).

In order to further explore the stability of protein-ligand interaction, molecular dynamics (MD) simulations were carried out on two kinds of protein-ligand complexes: SCN5A-Kaempferol and SCN5A-Oridonin. The root mean square deviation (RMSD) value is used to evaluate whether the simulation system has reached a stable state. The RMSD value within 1 nm indicates the relative stability of protein-ligand interaction in a physiological environment. As shown in Figure 3G, the RMSD values of the two complexes quickly stabilized at 0.75 ± 0.17 nm and 1.00 ± 0.19 nm, respectively. Root mean square fluctuation (RMSF) analysis confirmed the flexibility of amino acid residues, and the overall fluctuation of the system was not significant, which were 0.41 ± 0.26 and 0.34 ± 0.22, respectively. The radius of gyration (Rg) was analyzed to evaluate the tightness of receptor-ligand binding. As shown in Figure 3G, the Rg value of the complex remained stable during the whole simulation process, which was 1.84 ± 0.13 and 1.68 ± 0.11, respectively. The number of hydrogen bonds reflected the strength of protein-ligand binding, and SCN5A-Oridonin showed high hydrogen bond density and strength. These results indicated that Kaempferol and oridonin have good data performance in molecular docking and molecular dynamics simulation.

Based on these findings, we concluded that KA and ODN might bind to SCN5A and exert therapeutic effects on ARVC with PKP2 mutations.

### 3.4 KA exerts therapeutic effects on ARVC in cardiac organoids by binding to SCN5A

**FIGURE 4:**
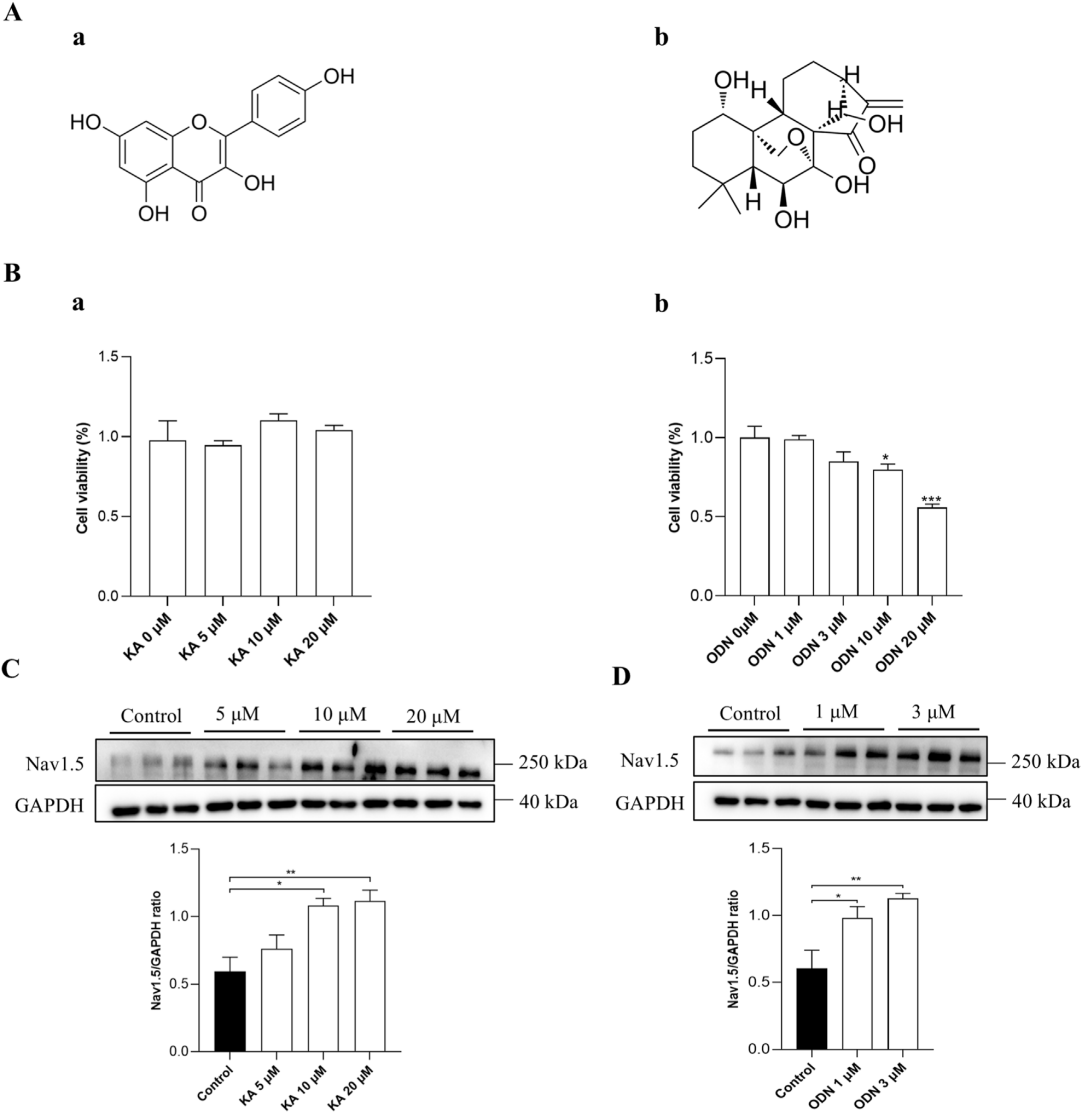
Verification of the screening model. **(A)** The chemical structures of KA **(a)** and ODN **(b)**. **(B)** Results of CCK-8 assays in PKP2^+/−^ cardiac organoids treated with KA **(a)** or ODN **(b)** at different doses. C and D, Western blot analysis of PKP2^+/−^ cardiac organoids treated with different doses of KA **(C)** or ODN **(D)**. Representative immunoblots of Nav1.5 are shown above each panel. The quantification of Nav1.5 protein levels is provided. One-way ANOVA followed by the Dunnett test was used for data analysis. Results are presented as the percentage of means ± S.E.M. (n ≥ 3). *P < 0.05, **P < 0.01, ***P < 0.005 vs. Control group.

**FIGURE 5:**
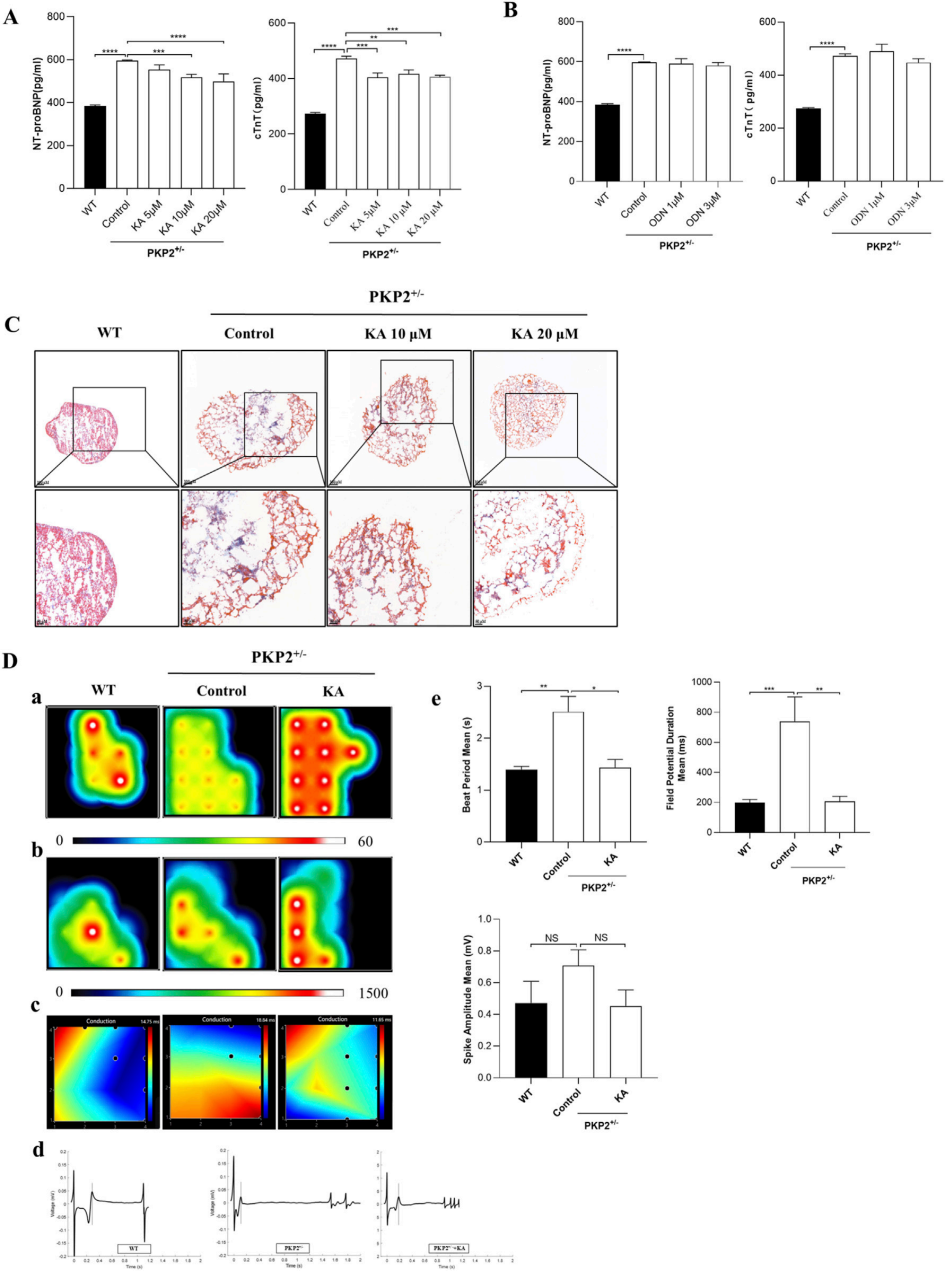
Functional validation of potential compounds. **(A,B)**, The anti-heart failure effects of different compounds in PKP2^+/−^ cardiac organoids. The expression levels of NT-proBNP **(A)** and cTnT **(B)** were detected by ELISA according to the manufacturer’s instructions. Cardiac organoids were treated with KA or ODN for 48 h. Then, the supernatant was collected and quantitated by BCA assay. **(C)** Results of Masson’s staining from WT and PKP2^+/−^ cardiac organoids. Scale bars, 100 μm. **(D)** Results of myocardial electrophysiology in cardiac organoids by using MEA. Typical heatmap of the excitatory firing rate **(a)**, spike amplitude **(b)**, and conduction **(c)** in WT or PKP2^+/−^ cardiac organoids. Visualization of field potential **(d)**, assessment of Beat Period Means, Spike Amplitude, and Field Potential Duration **(e)**. One-way ANOVA followed by the Dunnett test was used for data analysis. The results are presented as the percentage of means ± S.E.M. (n ≥ 3). *P < 0.05, **P < 0.01, ***P < 0.005 vs. Control group, NS, not significant.

To evaluate the therapeutic effect of KA and ODN on ARVC, Western blot, ELISA, MEA, and MASSON staining were conducted in the WT and PKP2^+/−^ cardiac organoids. The chemical structures of KA and ODN were shown in Figure 4A. Both KA and ODN increased the expression of SCN5A in a dose-dependent manner with no effect on cell viability in PKP2^+/−^ cardiac organoids (Figures 4B–D). Since heart failure is a distinct end-stage symptom of ARVC (Lu et al., 2022), the expression of cTnT and NT-proBNP was measured using the ELISA kit following pre-incubation with KA or ODN for 2 days. The results showed that KA reduced the expression of heart failure markers in a dose-dependent manner, whereas no such effect was observed with ODN (Figures 5A,B). Next, Masson staining was performed on cardiac organoids to assess the impact of KA on cardiac fibrosis, revealing that KA alleviated cardiac fibrosis in ARVC, particularly at the dosage of 20 μΜ (Figure 5C). Furthermore, the MEA assay demonstrated that KA improved the electrophysiological function of cardiac organoids by normalizing the abnormal heart rate, shortening the prolonged QRS waveform, and increasing myocardial contractility (Figure 5D). Additionally, *in vivo* validation in PKP2^+/−^ rats corroborated these findings: KA (20 mg/kg/day, i.g.) can improve the wide QRS waveform and alleviate arrhythmia (Supplementary Figure S3).

Taken together, these results demonstrate that KA can exert therapeutic effects on ARVC.

## 4 Discussion

In this study, we dedicate our efforts to identifying potential compounds targeting sodium channel proteins that may have therapeutic effects for treating ARVC. Using proteins analyzed from the ScRNA-seq and proteomics data of ARVC rats and their corresponding PPI networks, we constructed 27 machine learning (ML) models to predict binding affinities (BA) during the screening process. KA and ODN were predicted to bind to SCN5A with high affinity. To validate these predictions, we performed Western blot, ELISA, MEA, and Masson’s staining on cardiac organoids to assess the effects of these compounds. Our functional experiments revealed that KA alleviated heart failure symptoms, reduced cardiac fibrosis, and improved cardiac electrophysiological function in ARVC cardiac organoids. To the best of our knowledge, KA is the first compound identified to show therapeutic potential for ARVC.

While ARVC is classically attributed to desmosomal mutations, emerging evidence implicates voltage-gated sodium channel dysfunction, particularly SCN5A-encoded Nav1.5, as a critical co-driver of disease progression. The sodium channel dysregulation operates not only as a phenomenon of ARVC but as a central amplifier of electro-pathology. Focusing on this target represents a promising strategy to break the arrhythmia-fibrosis cycle (Cerrone and Delmar, 2014; Shaw, 2013; Delmar, 2012; Cerrone et al., 2012). Significantly, our finding that KA restores Nav1.5 expression in PKP2^+/−^ organoids, positioning sodium channel repair as a therapeutic way for ARVC management.

Recent studies establish KA as a multi-mechanism cardioprotective agent, modulating critical pathways, such as Nrf2, NF-κB, AKT/Bcl-2, and PI3K/Akt/GSK-3β. These interactions attenuate cardiac pathology through suppressing oxidative stress and inflammation, reducing myocardial collagen deposition, and inhibiting cardiomyocyte apoptosis. Additionally, KA preserves mitochondrial function and calcium homeostasis (Hua et al., 2022; Kamisah et al., 2023; Zhang et al., 2022). Critically, our data extend these findings to ARVC pathogenesis, demonstrating KA significantly reduces apoptosis in PKP2^+/−^ cardiac organoids (Supplementary Figure S5). Although β-blockers remain conventional ARVC therapy, they lack KA’s polypharmacological profile, and exhibit off-target and drug discontinuation effects (Jacobson et al., 2016). KA thus emerges as a promising multi-target therapeutic candidate addressing both fibrosis and arrhythmogenesis in ARVC.

In natural language processing (NLP), transformer-based models have demonstrated exceptional capabilities in understanding and generating human language (Vaswani, 2017). Recently, this architecture has been applied to computational chemistry, particularly in the analysis of SMILES strings, enhancing the prediction of molecular properties (Anonymous, 2019). Thus, in our study, we employed the SMILES Transformer with sequence-to-sequence capabilities of language models to efficiently capture the structural features of molecules. Compared to traditional graph-based neural networks, sequence-to-sequence models offer greater flexibility in handling the diversity of molecular structures, thereby enhancing both the efficiency and accuracy of feature extraction (Keneshloo et al., 2020). Additionally, we leveraged the ChEMBL database, which contains a vast collection of bioactive compounds and their target information, to pre-train the SMILES Transformer (ST) models, significantly improving the model’s generalization ability (Nowotka et al., 2017).

By using randomly sampled, unlabeled SMILES data as pre-training input, we enhanced the model’s predictive power for unknown compounds and mitigated overfitting. The 1024-dimensional molecular fingerprints derived from the maximum pooling output of the Transformer model provide a compact and accurate representation of molecular structures in lower dimensions, reducing information loss and improving the performance of subsequent classification models, such as GBDT. As a powerful ensemble learning technique, GBDT is particularly effective in handling complex nonlinear relationships and is well-suited for large-scale drug screening datasets (Zhang and Jung, 2021). Combining these models not only increases prediction accuracy but also makes the drug screening process more efficient and reliable.

In drug screening, the cardiac organoid model offers a more biologically relevant environment compared to traditional cell culture and animal models, providing significant advantages in efficacy verification (Chen X. et al., 2023). First, cardiac organoids can replicate the pathophysiological characteristics of heart disease at the microscopic level by simulating the three-dimensional structure and complex intercellular interactions of the human heart. This enhances their applicability in drug screening and mechanistic research, making them more physiologically relevant (Monteduro et al., 2023). Second, compounds identified via ML modeling can be more accurately validated for efficacy using cardiac organoid models. These models could not only demonstrate the interaction between drugs and their targets, but also capture the complex effects of drugs on cardiac function, such as myocardial contraction, signal transduction, and electrophysiological characteristics (Figures 5D). By integrating organoid model validation, prospective errors in drug development can be minimized, thereby improving the success rate of candidate compound screening (Zhu et al., 2024).

While our study establishes a novel BA prediction framework, two key limitations warrant acknowledgment. First, the absence of rigorous binding validation, such as Cellular Thermal Shift Assay (CETSA) or Microscale Thermophoresis (MST), leaves compound-target interactions reliant on computational evidence. Second, our experimental validation was constrained to eight prioritized candidates, reflecting the proof-of-concept scale of this work. Nevertheless, this approach successfully identified sodium channel binders with therapeutic potential in ARVC models, establishing a scalable platform for future large-scale compound screening.

In summary, this study presents a robust tool for drug discovery by integrating advanced deep learning with traditional ML methods, ensuring high performance across diverse molecular types and data scales. By integrating ML modeling with organoid validation, traditional screening methods are enhanced by modern biological models, significantly improving the accuracy and efficiency of drug screening processes, with broad application prospects.

## 5 Conclusion

Our machine-learning-based platform offers a novel approach for identifying potential compounds to treat ARVC. By integrating ML modeling with modern biological models, such as organoid validation, our platform provides valuable assistance in addressing the public health challenges posed by ARVC. KA shows promise as a lead compound for ARVC treatment.

## Data Availability

The original contributions presented in the study are publicly available. This data can be found here: NCBI Sequence Read Archive (SRA) under BioProject accession number PRJNA1292968; ProteomeXchange under dataset identifier PXD066271.

## Abbreviations

AAV, adeno-associated virus; ARVC, arrhythmogenic right ventricular cardiomyopathy; BA, binding affinity; CETSA, Cellular Thermal Shift Assay; ECG, electrocardiogram; GBDT, gradient-boosting decision tree; hiPSCs, human induced pluripotent stem cells; IF, Immunofluorescence; KA, Kaempferol; MEA: multiple microelectrode arrays; ML, machine learning; MRI: Magnetic Resonance Imaging; MST, Microscale Thermophoresis; NLP, natural language processing; ODN, Oridonin; PBMCs, peripheral blood mononuclear cells; PFA, paraformaldehyde; PPI, protein-protein interaction; PKP2, plakophilin 2; RMSD: root mean square deviation; scRNA-seq, Single-cell RNA-sequence; ST, SMILES Transformer; ROCK I, rho kinase inhibitor; UMAP, Uniform manifold approximation and projection.
