# Supplementary material for: Machine learning analysis of ARVC informed by sodium channel protein-based interactome networks
Source: Front Pharmacol. 2025 Jul 23;16:1611342. doi: 10.3389/fphar.2025.1611342 (PMC12326074; doi:10.3389/fphar.2025.1611342)
Supplement: Supplementary file 3 [file DataSheet1.pdf]

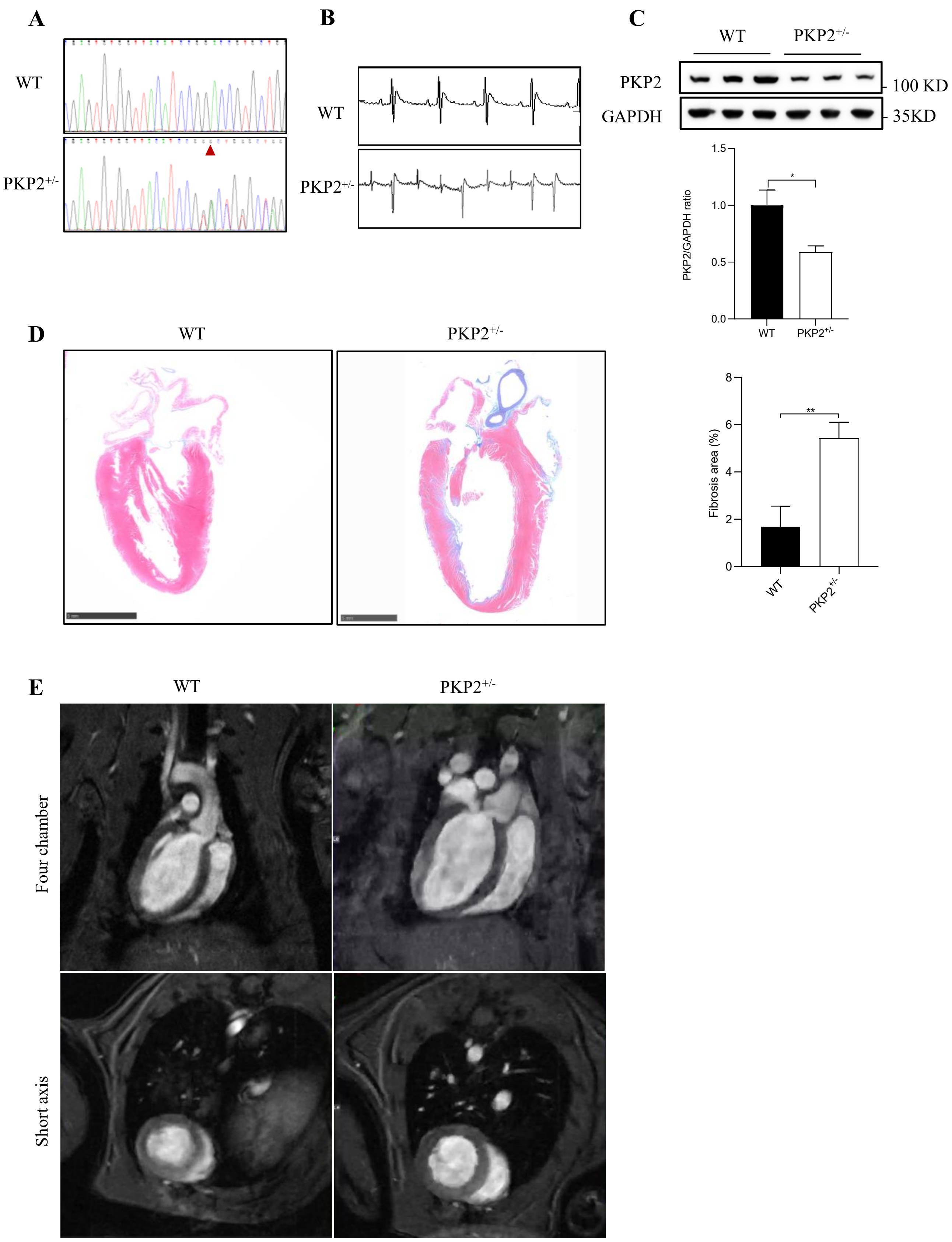

Supplementary Fig. 1. PKP2$^{+/-}$ rat model recapitulates cardiac physiological disease characteristics of ARVC. A, DNA Sequence of WT and PKP2$^{+/-}$ rats' hearts, . B, Representative ECG tracings from WT and PKP2$^{+/-}$ rats at 30 weeks. C, Western blot analysis of cardiac tissues from WT and PKP2$^{+/-}$ rats. D, Representative cardiac sections stained with Masson's staining and fibrosis quantification from WT and PKP2$^{+/-}$ rats at 30 weeks. Scale bars, 5 mm. The results are presented as the percentage of means ± S.E.M. (n = 3). Two-tailed unpaired t-test. $^{*}P < 0.05$, $^{**}P < 0.05$ vs Control group. E, Representative cardiac four-chamber and short-axis views from MRI in WT and PKP2$^{+/-}$ rats at 30 weeks.

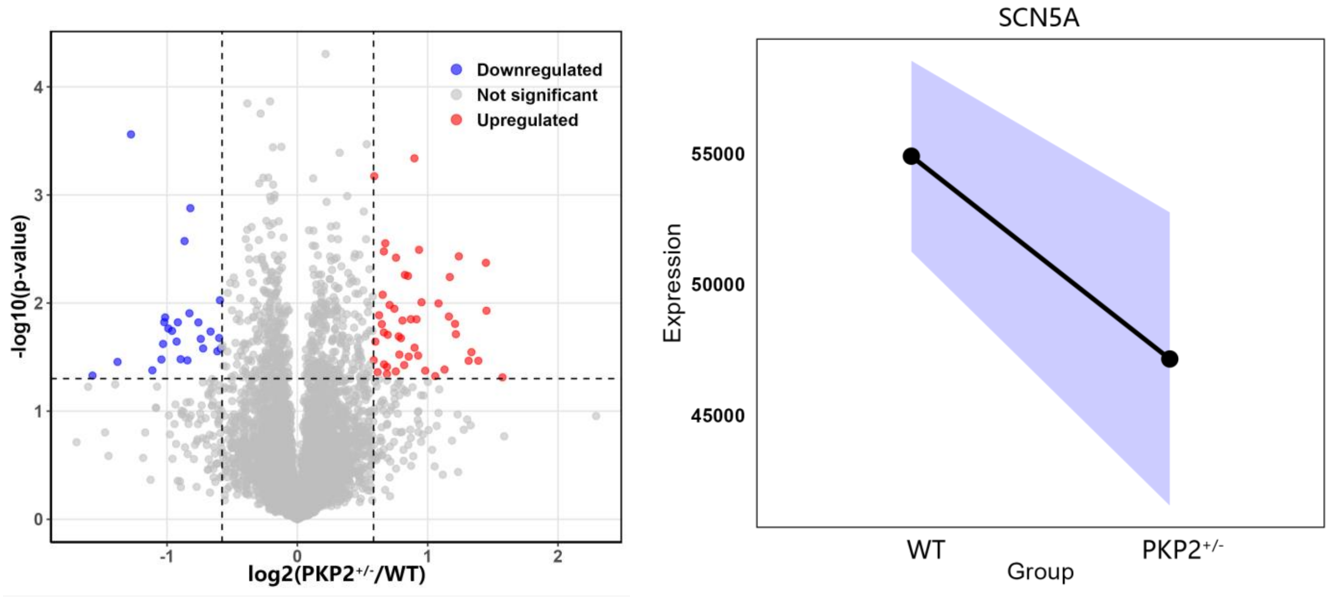

Supplementary Fig. 2. Proteomics of WT and PKP2$^{+/-}$ rats' hearts. A, Volcano plot of all of the expressed proteins from WT and PKP2$^{+/-}$ group (n = 4). B, Expression of SCN5A protein in WT and PKP2 PKP2$^{+/-}$ group.

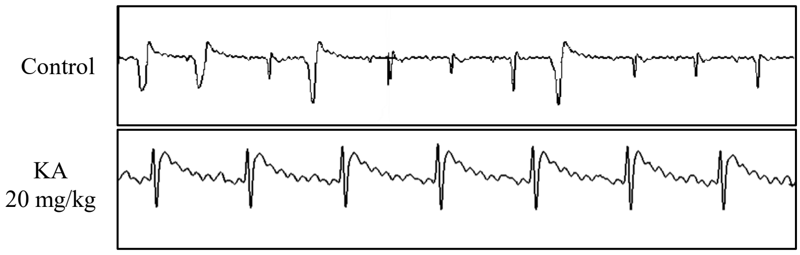

Supplementary Fig. 3. Representative ECG tracings from PKP2$^{+/-}$ rats (> 30 weeks) treated with KA 20 mg/kg/day i.g. or vehicle control for 10 days.

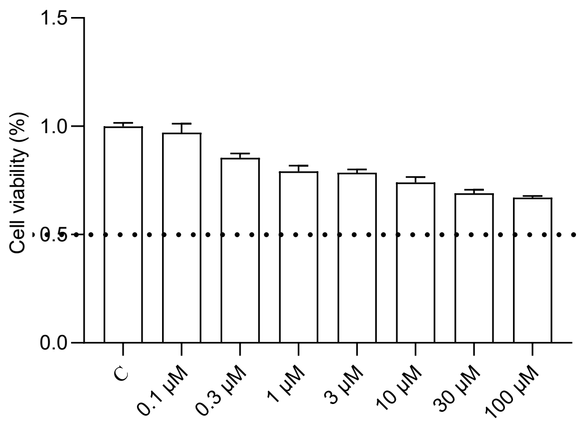

Supplementary Fig. 4. Results of CCK-8 assays in AC16 treated with KA at different doses. Results are presented as the percentage of means ± S.E.M. (n ≥ 6).

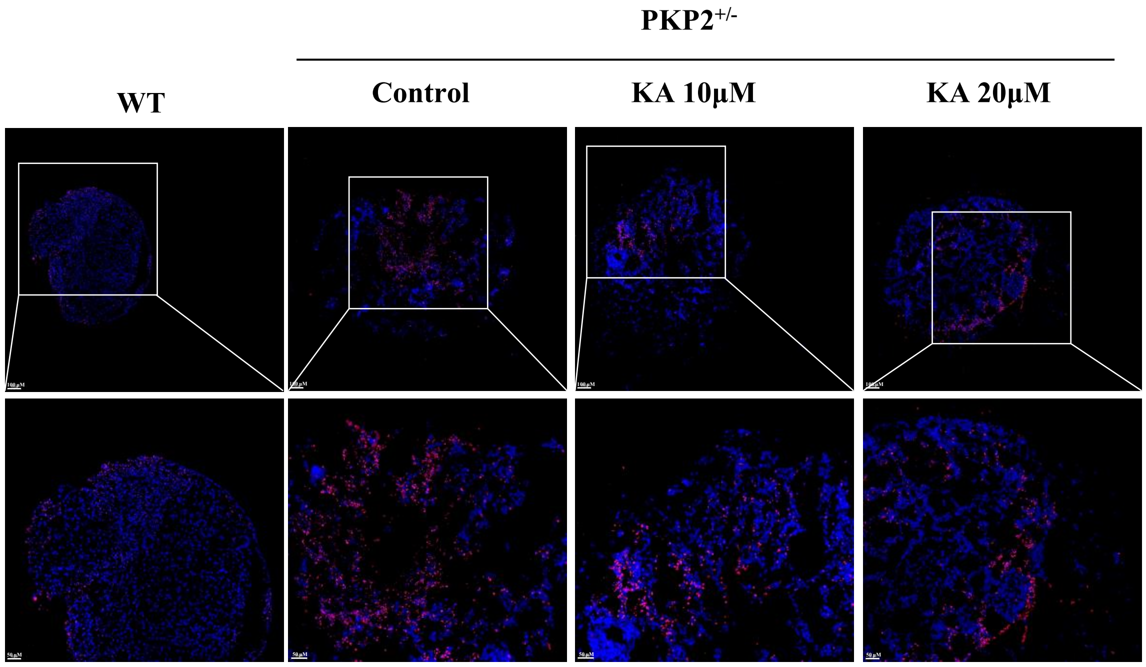

Supplementary Fig. 5. TUNEL assay of KA in WT and PKP2$^{+/-}$ cardiac organoids.
